# Supplementary material for: MetaRibo-Seq measures translation in microbiomes
Source: Nat Commun. 2020 Jun 29;11:3268. doi: 10.1038/s41467-020-17081-z (PMC7324362; doi:10.1038/s41467-020-17081-z)
Supplement: Supplementary file 10 — Supplementary Data 7 [file 41467_2020_17081_MOESM10_ESM.zip › File2/Confidence_High_Taxonomy/321672_out.krona.html]

Javascript must be enabled to view this page.

members
magnitude
magnitudeUnassigned
count
unassigned
taxon
rank

321672\_out

9

2
superkingdom
9

1239
9
phylum

9
class
186801

186802
order
9

9
family
31979

1485
genus
9


SRS015782\_contig\_number\_2238SRS146812\_contig\_number\_58851
1896983
species
2

1
species
2293035

SRS018623\_contig\_number\_6994

1262836

SRS014613\_contig\_number\_16658SRS015854\_contig\_number\_contig-100\_11.103031SRS016095\_contig\_number\_3521SRS063040\_contig\_number\_36442SRS098571\_contig\_number\_48466SRS144135\_contig\_number\_25147
6
species
